# The Effects of Three-Dimensional Ligand Immobilization on Kinetic Measurements in Biosensors

**DOI:** 10.3390/polym14020241

**Published:** 2022-01-07

**Authors:** Elisa Chiodi, Allison M. Marn, Monireh Bakhshpour, Nese Lortlar Ünlü, M. Selim Ünlü

**Affiliations:** 1 Department of Electrical Engineering, Boston University, Boston, MA 02215, USA; ammarn@bu.edu (A.M.M.); myucel@bu.edu (M.B.); nunlu@bu.edu (N.L.Ü.); 2 School of Engineering, Computing, and Construction Management, Roger Williams University, Bristol, RI 02809, USA; 3 Department of Chemistry, Hacettepe University, Ankara 06800, Turkey; 4 Department of Biomedical Engineering, Boston University, Boston, MA 02215, USA

**Keywords:** surface chemistry, label-free biosensing, molecular affinity, three-dimensional polymeric matrix

## Abstract

The field of biosensing is in constant evolution, propelled by the need for sensitive, reliable platforms that provide consistent results, especially in the drug development industry, where small molecule characterization is of uttermost relevance. Kinetic characterization of small biochemicals is particularly challenging, and has required sensor developers to find solutions to compensate for the lack of sensitivity of their instruments. In this regard, surface chemistry plays a crucial role. The ligands need to be efficiently immobilized on the sensor surface, and probe distribution, maintenance of their native structure and efficient diffusion of the analyte to the surface need to be optimized. In order to enhance the signal generated by low molecular weight targets, surface plasmon resonance sensors utilize a high density of probes on the surface by employing a thick dextran matrix, resulting in a three-dimensional, multilayer distribution of molecules. Despite increasing the binding signal, this method can generate artifacts, due to the diffusion dependence of surface binding, affecting the accuracy of measured affinity constants. On the other hand, when working with planar surface chemistries, an incredibly high sensitivity is required for low molecular weight analytes, and furthermore the standard method for immobilizing single layers of molecules based on self-assembled monolayers (SAM) of epoxysilane has been demonstrated to promote protein denaturation, thus being far from ideal. Here, we will give a concise overview of the impact of tridimensional immobilization of ligands on label-free biosensors, mostly focusing on the effect of diffusion on binding affinity constants measurements. We will comment on how multilayering of probes is certainly useful in terms of increasing the sensitivity of the sensor, but can cause steric hindrance, mass transport and other diffusion effects. On the other hand, probe monolayers on epoxysilane chemistries do not undergo diffusion effect but rather other artifacts can occur due to probe distortion. Finally, a combination of tridimensional polymeric chemistry and probe monolayer is presented and reviewed, showing advantages and disadvantages over the other two approaches.

## 1. The Importance of Anti-Fouling Materials for Label-Free Kinetics

Label-free binding kinetics is essential to describe the interaction mechanisms of proteins, antibodies, oligonucleotides, and more [1]. Differently from labeled or end-point methods, label-free kinetics allows for real time investigation of binding reactions, removing the constraint of having a third interacting element (the label) [2,3]. The possibility of studying the behavior of a wide range of bio-agents in their native conformation is attractive for a number of reasons, especially for the field of drug and antibody development. Label-free kinetic characterization of a drug-receptor interaction is the least artificial way to measure the concentration-dependent response of the ligands to the compound [3,4].

The label-free sensors currently available on the market enable the investigation of hundreds of probes at a time against a single antigen [5], as well as a single probe against multiple targets [6]. The dynamic range of the available technology is incredibly large, both in terms of analyte molecular weight (MW) and concentration. Nowadays, novel sensors can handle analytes as tiny as small molecules (MW<1 kDa) [4,5,7,8] and as large as whole cells [9], in the femtomolar to millimolar range (fM-mM).

One of the main limitations of label-free kinetics measurements is the requirement to immobilize one of the two interacting agents (the ligand) onto the sensor surface, in order to measure the amount of target analyte binding to the ligands. Consequently, the surface of the sensor needs to be chemically activated and functionalized by coating it with a specifically designed material containing reactive groups that can stably anchor molecules, without permanently denaturating their structure [10]. Moreover, such material needs to attach the probe molecules to form a high density layer while otherwise being repulsive to other non-specific interactions (anti-fouling). Achieving stable, high density immobilization of biomolecules is the main goal of the field of surface chemistry for biosensors, which has seen significant development in the past few years due to the increasing need for dedicated materials with very specific features: the ideal functionalized surface needs to have all the characteristics enumerated above and, moreover, it needs to be customizable, in order to adapt to different sensor materials. While most sensors are made of glass (silica) or glass-like materials (hard plastics), some of them feature a metallic component. For example, surface plasmon resonance (SPR) sensors, the current standard in label-free kinetics, base their working principle on a thin layer of gold which coats a glass prism. Hence, SPR-dedicated surface chemistry needs to adapt to an unusual, impractical chip material [11].

Finding the perfect balance of high probe density and low steric hindrance, all while avoiding non-specific adhesion and molecular distortion, is non trivial. Three dimensional ’soft’ polymeric coatings such as carboxymethyl dextran matrices allow to maintain the native probe conformation, without impacting the biofunctionality of the molecule [12]. In some cases, multilayer probe immobilization is also provided, to increase surface probe density without causing steric hindrance. However, this approach presents a number of disadvantages, one above all, the reaction will most likely suffer from mass transport limitations due to the slow diffusion of analytes inside the hydrogel [13,14]. In the past decade, many anti-fouling soft polymers have been developed which focus on maintaining high probe density while reducing mass transport limitation, non-specific binding, and steric hindrance. Most of those approaches utilize *semi-tridimensional* materials, which form a very thin layer (<10 nm) on the surface, while still containing a soft backbone that creates a thin 3D structure.

Here, we will give a brief overview of the most widely utilized methods of molecular immobilization for label-free kinetics, mostly highlighting the effect of tridimensionality of polymeric matrices on sensors’ sensitivity enhancement and analyte diffusion. As better explained in Section 2, Section 3 and Section 4, various approaches are nowadays employed, most of which rely on soft polymers and hydrogels, while for some applications a simple silane-based surface activation is sufficient (Section 3). This commentary would like to focus on the main differences, advantages and disadvantages of these techniques.

## 2. CM-Dextran Matrices for SPR Biosensors

SPR is a label-free optical sensing tool which relies on resonant coupling of an evanescent field with the plasmonic excitation of the electrons in a gold layer to measure local refractive index variations. SPR biosensors provide real-time monitoring of interactions of various biochemical analytes, enabling fast biomolecular kinetics measurements with high specificity and sensitivity without labeling. In the past decade, SPR platforms have been utilized for the detection of viruses, bacteria, biosimilar molecules and proteins. Studies show that accurate and early recognition of infectious diseases biomarkers can be performed in real-time [15]. Today, these biosensors are utilized in many areas, including biochemical studies such as investigation of protein–protein or DNA–DNA interactions, in clinical, environmental, and agricultural settings [16].

In practice, an SPR biosensor consists of biological recognition units (probes) immobilized on top of a gold-coated glass prism, while a solution containing target molecules is flowed across the functionalized surface through a fluidic chamber. Monochromatic p-polarized light is shined at a specific angle $\theta_{SPR}$ on one side of the prism, and it propagates from a medium with higher refractive index ($n_{glass}$) to a medium with lower refractive index ($n_{solution}$). In this configuration, total internal reflection (TIR) can be achieved for all incidence angles above a critical angle $\theta_{c}=\sin^{-1}\left(n_{solution}/n_{glass}\right)$. Since $\theta_{SPR}>\theta_{c}$, this generates an evanescent field which extends into the solution with exponentially-decaying amplitude. When phase-matching conditions are met, the evanescent field is strongly absorbed by the ≈50 nm-thick gold layer [11]. The specific angle of incidence $\theta_{SPR}$ which meets the phase-matching conditions depends very strongly on the refractive index in proximity of the gold layer. By imposing phase matching at the interfaces, one can derive the optimal incidence angle $\theta_{i}=\theta_{SPR}$ to obtain resonant coupling of the evanescent field with the plasmon excitation [11,17,18]. This angle is very sensitive to refractive index changes in the proximity of the sensor surface within the penetration depth of the evanescent field. Thus, by monitoring the variations of the angle where the dip is detected, it is possible to discriminate very slight refractive index changes close to the gold surface, ideally enabling very sensitive detection of biomolecular binding.

However, since the evanescent field extends for hundreds of nanometers into the solution, one of the main disadvantages of SPR is its tremendous sensitivity to changes in the solution refractive index and other environmental factors such as, for example, temperature or pH variations. Choosing the best buffer solution for SPR measurements is difficult, especially when dealing with small molecules that are not soluble in aqueous solvents. In most cases, dimethyl sulfoxide (DMSO) is the preferred solvent to dissolve low-molecular weight compounds. However, on SPR sensors, a change in refractive index due to the presence of 1% DMSO causes a background signal corresponding to as much as hundred times the signal generated by low molecular weight molecules binding [19]. In order to minimize this effect, many different approaches have been developed, most of them focusing on the surface functionalization on the sensor chip. Since the gold-based sensor was first introduced, SPR-tailored surface chemistry has evolved immensely. Recently, SPR biosensors have been applied to the detection of many diverse target molecules by using various immobilization technologies, such as aptamer-based, immunoaffinity, chemical affinity, and covalent bonding [15]. The most common of these is covalent bonding of molecules inside a thick, hydrogel-like carbomethylated dextran (CM-dextran) matrix.

**Figure 1:**
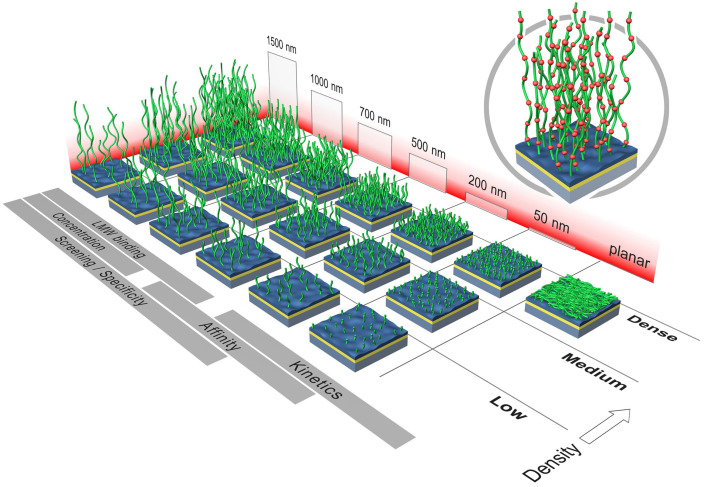
SPR chips commercially available from Xantec, Inc. [22] (© Xantec 2021, all rights reserved).

The early attempts at probe immobilization for SPR were based on a single layer of thiolated groups, and naturally produced a monolayer of biomolecules on the gold surface. However, as the need for investigating smaller molecules increased, both a higher density of probes, as well as minimization of the solvent effect (‘bulk’ effect) was necessary, in order to provide sensitivity to perform small molecule affinity measurements. One approach that proved successful in terms of balancing high probe density and low steric hindrance was the development of three dimensional probe immobilization in thick hydrogel matrices. Specifically designed for SPR sensors, CM-dextran is a carbohydrate polymer that creates a tridimensional structure by adding 50–1500 nm of thickness to the sensor surface, allowing for most of the evanescent wave penetration depth to be filled with probe molecules. This reduces the bulk effect while increasing the effective volume where target molecules can bind and generate a signal [20,21]. The number of probes per unit volume can also be tuned, by having different hydrogel densities, as shown in Figure 1.

The approach of covalently immobilizing probe molecules inside a polymeric matrix solves some of the issues of SPR sensors; however, it also introduces non-negligible artifacts. Mainly, the fact that the probe molecules are submerged in a thick solid matrix limits the kinetics of the reaction, leading to mass transport limitation [13]. The inherently slow diffusion through the hydrogel limits the velocity at which the solution is replaced by the flow. The reaction is therefore mostly diffusion limited, which in turn means that the measured binding rate is actually the volumetric diffusion rate. Moreover, the molecules that are immobilized towards the top of the matrix are more likely to bind, since the target molecules have a higher chance of interacting with them, while the probes that are submerged deeper and closer to the gold surface are less likely. Moreover, given the exponential-decaying nature of the evanescent wave, the target molecules binding closer to the gold layer generate a bigger signal with respect to those binding further away from the surface. This creates further confusion when interpreting SPR data, causing a discrepancy in the calculated association and dissociation constants.

In order to address some of these issues, available fitting software for SPR data such as EvilFit [14,23] or Scrubber [24] provide various binding models, some of which can take mass transport limitation into account when calculating on and off rates, by considering first-order corrections to the fitting model. In this first-order approximation, the flow channel is treated as a two compartment system, where a *depletion zone* is defined as the section of the channel close to the gold surface, containing the dextran matrix and extending slightly into the solution. When mass transport limitation is present, the concentration of analyte inside the depletion zone is lowered due to binding, and the on and off rates are affected by this change in concentration, causing the reaction to be diffusion limited [13]. More specifically, if mass transport limitation were not present, the surface binding of a single class of analytes to a single class of ligands (1:1 interaction) would follow a simple rate equation:

(1) $$\frac{dS}{dt}=k_{ON}C\left(S_{max}-S\right)-k_{OFF}S$$

where *S* is the measured binding signal, *C* is the concentration of the analyte, $S_{max}$ is the maximum achievable binding-corresponding to the saturation of available surface binding sites, $k_{ON}$ and $k_{OFF}$ are the on and off rates of the reaction, defining its affinity. The solution of the differential equation yields an analytical solution, which can be differentiated between the association phase (binding in presence of the analyte in solution) and dissociation phase (separation of the molecular complex in absence of the analyte). The two equations can be written as [14]:

(2) $$S\left(t\right)=\left\{\begin{array}{cc} 0 & 0<t<t_{0}\\ S_{eq}\left(1-e^{-\left(k_{ON}C+k_{OFF}\right)\left(t-t_{0}\right)}\right) & t_{0}<t<t_{1}\\ S_{eq}\left(e^{-k_{OFF}\left(t-t_{1}\right)}\right) & t>t_{1} \end{array}\right.$$

where $t_{0}$ is the analyte injection time, and $t_{1}$ is the starting time of the dissociation phase, where the analyte solution is substituted with wash buffer. The binding at equilibrium is an isotherm of the form [23]:

(3) $$S_{eq}=\frac{S_{max}}{1+\frac{K_{D}}{C}}$$

where $K_{D}=k_{OFF}/k_{ON}$ represents the dissociation constant, also defined as the *equilibrium constant*. Now, the two compartment model considers a depletion of the analyte molecules in the volume close to the surface, resulting in a variation of the concentration from the value *C* to a lower ‘depleted’ value $C_{d}<C$, which is not constant, but rather changes with time, as the surface binding sites start to saturate:

(4) $$\frac{dC_{d}}{dt}=k_{TR}\left(C-C_{d}\right)-\sum_{i=1}^{N}\frac{dS_{i}}{dt}$$

For *N* binding sites considered, i=1,⋯,N. Here, the transport rate parameter ($k_{TR}$) is introduced, which has been demonstrated to approximately depend on the diffusion coefficient *D* of the analyte as:

(5) $$k_{TR}\approx 1.282v^{1/3}hl^{-1/3}D^{2/3}$$

where *v* is the flow rate, *h* and *l* are the height and length of the flow channel, respectively. Therefore, Equation (1) mentioned above assumes the form:

(6) $$\frac{dS_{i}}{dt}=k_{ON,i}C_{d}\left(S_{max}-S_{i}\right)-k_{OFF,i}S_{i},$$

which in turn produces distinct association and dissociation rates for each binding site. Despite this being a first-order approximation, thus still not representative of the real course of events, applying this correction is obviously far from trivial and requires huge computational efforts, as well as producing complex results. Alternatively, TraceDrawing software [25], another popular SPR data analysis software, chooses to approximate the discrete increment of bound analyte to single probes to a continuous function, in order to obtain single $k_{ON}$, $k_{OFF}$ and $k_{TR}$ values by numerically solving the following system of differential equations for S(t):

(7) $$\left\{\begin{array}{c} dS/dt=(k_{ON}C_{d}\left(t\right)-k_{OFF})S\left(t\right)\\ dC_{d}/dt=k_{TR}\left(C-C_{d}\left(t\right)\right)-dS\left(t\right)/dt\\ S\left(t=0\right)=0;S\left(t=t_{sat}\right)=S_{max} \end{array}\right.$$

where $t_{sat}$ is the time where all binding sites are saturated, and the maximum reachable signal is measured ($S_{max}$) [25].

The presence of the CM-dextran layer poses an additional complication: the probes that are immobilized on top-closer to the solution-have indeed a higher probability to be reached by the target molecules, which will initially mostly bind in that region, forming a superficial layer. When the probe density in the layer is really high, such as for small molecule characterization, the generated steric hindrance will further inhibit the binding to the probes close to the gold surface. Therefore, both steric hindrance and diffusion affect binding to the deeper molecules, reducing the probability of saturation of all binding sites. Additional corrections to the model are, therefore, necessary, and *depleted models* are available in the TraceDrawing software, which consider the dependency of the maximum reachable signal $S_{max}=S_{max}\left(t,C_{d}\right)$ on the presence of increasing amounts of bound analyte [25].

This discussion shows that having a three-dimensional distribution of probes is not an ideal solution to compensate for the lack of sensitivity of the biosensing platform. Instead, working with a monolayer of probes is desirable, but, in order to achieve that, a highly sensitive biosensor is needed, especially when trying to detect and characterize low molecular weight compounds (small molecules). As it can be observed in Figure 1, the suggested chemistry for SPR when working with low-molecular weight (LMW) analytes is the thickest available CM-dextran coating, while those suggested for kinetic measurements are significantly less dense. For all of the reasons outlined above, most data analysis programs require the user to provide measurements of $k_{ON}$ and $k_{OFF}$ at multiple concentrations, in order to be able to estimate an isotherm and interpolate for $K_{D}$ values. This obviously causes each experiment to be more time consuming, as well as requiring more sample. Moreover, the advantage of having real time determination of association and dissociation constants is lost when considering endpoint equilibrium values [11].

**Figure 2:**
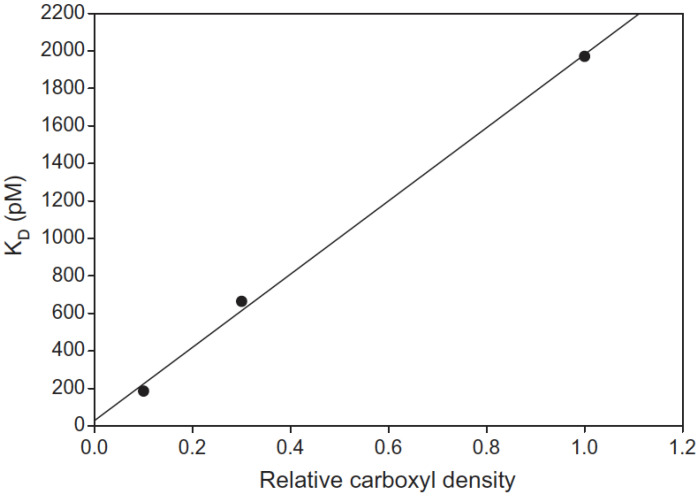
The effect of CM-dextran density on mAb/Ag affinity measurements. Reproduced with permission from [26]. Copyright 2012 Copyright Elsevier.

Additionally, it has been demonstrated that the density of the CM-dextran layer influences measured $K_{D}$ values due to electrostatic interactions [26]. The presence of carboxyl groups in the dextran matrix results in an overall negatively charged surface, and for the same analyte, multiple $K_{D}$ values are obtained when utilizing different CM-dextran chips due to a combination of steric hindrance and electrostatic repulsion. As an example, Drake et al. have shown the impact of the density of CM-dextran chips on the affinity constants measured for an antigen-antibody complex (Ag/mAb), as reported in Figure 2.

Overall, the performance of an SPR biosensor depends on both its optical characteristics as well as on the features of the surface modification [27]. It should be highlighted that the use of molecular imprinted technology presents advantages in stability and reproducibility, as widely described in the literature. To compensate for the lack of sensitivity in detecting low-molecular weight analytes, Matsui et al. have developed a gold nanoparticle-embedded molecularly imprinted polymer for sensitive and selective detection of small molecules on SPR sensors [28]. Moreover, Bereli et al. have utilized amine-functionalized gold nanoparticles captured by an anti-IgM-coated sensing surface of an SPR chip to perform reliable and sensitive detection of immunoglobulin M antibodies [29]. These systems represent an improvement over the standard SPR sensors in terms of sensitivity, however, they fail in part to comply with the principle of a label-free sensor due to the presence of gold nanoparticles.

This discussion highlights the compelling need for SPR-alternative biosensors that are able to produce reliable results for small molecule characterization, and various solutions have been proposed [4,30]. Among others, our group has recently developed a biosensing platform with small molecule sensitivity [5], which works with a single layer of probes immobilized on a polymeric coating [31], as better described in Section 4.

## 3. Epoxysilanization and SAM Monolayers

For label-free sensors not based on metal surfaces, the simplest and cheapest way to anchor molecules to a glass surface is to silanize the surface, in most cases by utilizing a bi-dimensional epoxysilane coating. Epoxysilane polymers, such as (3-glycidyloxypropyl)trimethoxysilane (GLYMO), have two functional ends which perform two different tasks: the silane groups bind covalently to plasma-activated silica surfaces by reacting with -OH groups, while the epoxy end reacts with amine groups in proteins or other amine-modified biomolecules, linking them to the surface.

The process of coating with epoxysilane polymers is both cost effective and relatively fast, it can be safely performed in any laboratory environment and does not require any particular expertise. Hence, it remains one of the most commonly utilized functionalization methods in biosensing applications, despite having some pretty significant disadvantages. One of the main reported issues when dealing with epoxide coatings is surface inhomogeneity [32] as well as spot-to-spot variation [33]. Coffee ring effects have also been observed in some cases [32], causing uneven probe distribution across the spot. The majority of these problems can be correlated to the nature of the immobilization reaction on epoxide surfaces. Coating a glass surface with GLYMO will form a self assembled monolayer (SAM) of around ≈2 nm in thickness [31,33] that will react with the molecules’ primary amine groups to anchor them to the surface. The high reactivity and bi-dimensionality of the SAM implies that the probes will be strongly attracted to the surface, to the point that their structure might be distorted and their epitopes could be inaccessible for binding. This issue is particularly relevant when working with protein probes. Proteins can indeed be classified as *soft proteins* and *hard proteins*, which refers to their inclination to denaturate. If a protein contains a large number of disulfide bonds, it will be more resistant to denaturation, and it will be classified as a hard protein. One example of a hard protein is bovine serum albimin (BSA), commonly used as a model for protein-binding assays, which contains seventeen disulfide bonds. On the other hand, α-lactalbumin is identified as a soft protein, containing only four disulfide bonds [12]. Immunoglobulin G (IgG) is also a soft protein: most antibodies belong to this category, which highlights the importance of being able to efficiently handle this type of probe. Immobilizing soft proteins is obviously more complicated, due to their delicate structure, which requires particular care when strongly attaching them to a surface. Epoxide coatings are usually not ideal in this case, due to their tendency to strongly ”pull” the molecule towards the surface from multiple sides, and flatten it, unintentionally promoting denaturation [34].

Therefore, SAM-based epoxide coatings have opposite disadvantages with respect to dextran matrices: the reaction is not limited by diffusion, as long as the flow speed is above a certain limit [13]. However, the measured binding affinity will be inconsistent if the probes are denaturated, inhomogenously distributed, or unable to bind. The lack of tridimensionality in this case is an issue. Additives can be incorporated into spotting solutions to improve probe distribution [32], but there is still not a consensus in the literature on how to solve the structural distortion problem. Similarly to the case of the mass-transport for dextran chemistries, a number of models have been developed which take into account the impact of structural deformation and probe inaccessibility of immobilized molecules with respect to in-solution hybridization [31,35].

## 4. Monolayer Polymeric Coatings: The Sweet Spot

Thus, far, we have described the two most popular surface functionalization methods, which are to this day utilized by numerous biosensing technologies, such as SPR and bio-layer interferometry (BLI) [36,37]. Both techniques have advantages and disadvantages; tridimensional thick hydrogel matrices such as carbomethyldextran for SPR have a limitation in terms of efficient analyte diffusion, while bi-dimensional coatings such as GLYMO tend to cause steric hyndrance, probe inhomogeneity distribution and molecular structure distortion. Therefore, surface tridimensionality appears to be critical in order to maintain probe structure, expose binding epitopes and avoid steric hindrance. At the same time, multi-layered immobilization in thick matrices causes discrepancy in probe availability to binding, where probes closer to the solution are more likely to bind with respect to those submerged deeper into the matrix.

**Figure 3:**
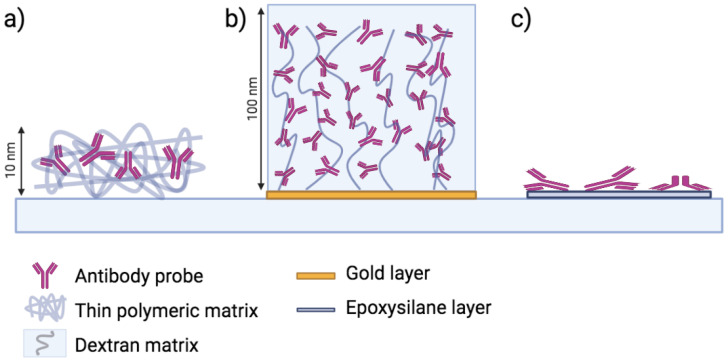
A graphical scheme of immobilized antibody probes on: (**a**) 10 nm thick polymeric coating (MCP-2); (**b**) 100 nm thick CM-dextran matrix; (**c**) epoxysilane (GLYMO).

As explained above, SPR has a real need for thicker coatings given its dependence on the refractive index changes throughout its penetration depth. However, most other optical sensors do not have this limitation, and would therefore benefit from a monolayer of probes but still require low steric hindrance along with intact, undeformed probes. Many polymeric surface coatings have been developed that have the ability to form a thin but tridimensional matrix on the sensor surface, maintaining probe structure and creating a monolayer of molecules equally available for binding. Most of these coatings are based on poly(ethylene)-glycol (PEG) [38,39]. Others are still based on surface silanization, but provide a more complex structure that creates an hydrophilic, anti-fouling thin polymeric matrix providing a monolayer of surface probes that maintain their native structure. One example is the co-polymer DMA-NAS-MAPS developed by Chiari et al. [40,41], commonly known as MCP-2. The polymer contains trimethoxysilane moieties which can stably bind to the oxide groups present on the silica surface, while the probes are covalently attached through an amide bond between the active succinimidyl esters groups and the probes’ free amine groups. The MCP-2 polymer has been utilized in numerous biosensing experiments, demonstrating its versatility [42,43]. Similar to epoxysilane, the coating process is rapid and does not require any particular laboratory equipment or expertise. Moreover, the polymer structure is customizable, and from MCP-2 a whole family of polymers was developed, ranging from click-chemistry polymers based on NHS esters, to fluoropolymers [44]. Moreover, it can be utilized to coat a variety of materials, including paper [45]. Our group has successfully shown the ability of MCP-2 to immobilize both hard and soft proteins [44], forming a layer of single probes which extend into the solution when the polymer is hydrated. The thickness of the polymer was estimated to vary from ≈2 nm in dry condition (similar to epoxysilane) to ≈10 nm when hydrated [31]. A schematic illustration of the difference between the three approaches (CM-dextran, epoxysilane and thin polymer) is represented in Figure 3.

Finally, the ability to work with a single layer of probes in label-free biosensing resides in the high sensitivity of the sensor. Our group has recently developed the Interferometric reflectance imaging sensor (IRIS), a highly sensitive, multiplexed label-free platform for binding kinetics studies [46,47,48]. With a huge dynamic range in analyte size ranging from small molecules [5] to extracellular vesicles [49], this technology has enabled kinetic characterization of hundreds of different molecules. The combination of the IRIS platform with the surface chemistry provided by the MCP polymers has produced significant results in molecular characterization and single particle detection [10,50,51,52,53] with minimal instrumentation requirements [54].

Still, this method certainly has some disadvantages as well: first of all, it is not the most cost-effective method, especially when compared to the very affordable epoxysilane. Depending on the application, tridimensionality might not be necessary, particularly when working with hard proteins, and in that case a cheaper solution might be preferred. Further, similarly to silanization, MCP-2 polymeric coating provides random orientation of the probes on the surface, potentially affecting probe activity by masking the antigen binding sites. In some cases, especially with antibody probes, the attachment of the probes to the sensor surface through multiple binding sites can inhibit antigen binding due to the steric hindrance caused by the presence of the surface and adjacent probe molecules. Thus, since we are listing here the features of the ideal surface chemistry, oriented immobilization would definitely be considered as a requirement, in order to expose all the available binding sites to the analyte solution, maximizing the binding signal [51].

## 5. Conclusions and Perspectives

To summarize, the compelling need for efficient characterization of many different analytes correlates with the development of robust surface chemistry strategies, which succeed in consistently immobilizing molecules on a variety of different surfaces. We have summarized the advantages and disadvantages of the methods described in this paper in Table 1. Three-dimensional coatings have the advantage of maintaining the native structure of the molecular probe. SPR-based sensors often employ CM-dextran matrices which allow for increased binding signal by multi-layering probes in a thick polymeric coating. This causes most binding reactions to be diffusion-limited due to the slow diffusion through the matrix, as well as reducing the maximum achievable signal due to steric hindrance. However, bi-dimensional coatings forming a single layer of probes can promote denaturation and influence the bioactivity of the probe.

Preserving the stability of the molecules is fundamental, and ideally a highly sensitive biosensor should be able to work with a single layer of biomolecules that have been efficiently immobilized on the sensor by using a semi-tridimensional surface chemistry. This has been done in multiple ways, and our group in particular has developed a label-free biosensor with small molecule sensitivity that works with a single layer of stably-immobilized antibody by utilizing an organic polymer that forms a 10 nm (hydrated) structure and preserves probes’ bioactivity. Another very interesting, novel approach has been introduced by Singh et al. [55], who have developed an innovative method for enhancing the sensitivity of SPR by immobilizing a single layer of molecules on a thin, graphene-based surface in a noncovalent manner.

Moreover, as explained above, an additional method for further improving the efficiency of the binding without having multiple layers of probes is to orient the molecules, as opposed to random immobilization, so that all molecules have their epitopes available for binding. Multiple strategies have been developed on this topic, including affinity-based methods, such as for example DNA-directed immobilization [51], and click-chemistry [43].

## Figures and Tables

**Table 1:** Main features and limits of the immobilization methods for label-free bioassays discussed here.

Immobilization Strategy	Type of Sensor	3D Structure	Diffusion Limitation	Probe Denaturation
Epoxysilane [33]	Most LF sensors	no	no	yes
Copoly(DMA-NAS-MAPS) [40]	IRIS platform	yes	no	no
Carboxymethyl dextran [21,56]	SPR sensors	yes	yes	no

## Data Availability

Not applicable.
